# Supplementary material for: Vitamin K2 (MK-7) attenuates LPS-induced acute lung injury via inhibiting inflammation, apoptosis, and ferroptosis
Source: PLoS One. 2023 Nov 27;18(11):e0294763. doi: 10.1371/journal.pone.0294763 (PMC10681318; doi:10.1371/journal.pone.0294763)
Supplement: S1 Raw images — (ZIP) [file pone.0294763.s001.zip › Original image for checking.pptx]

## Slide 1
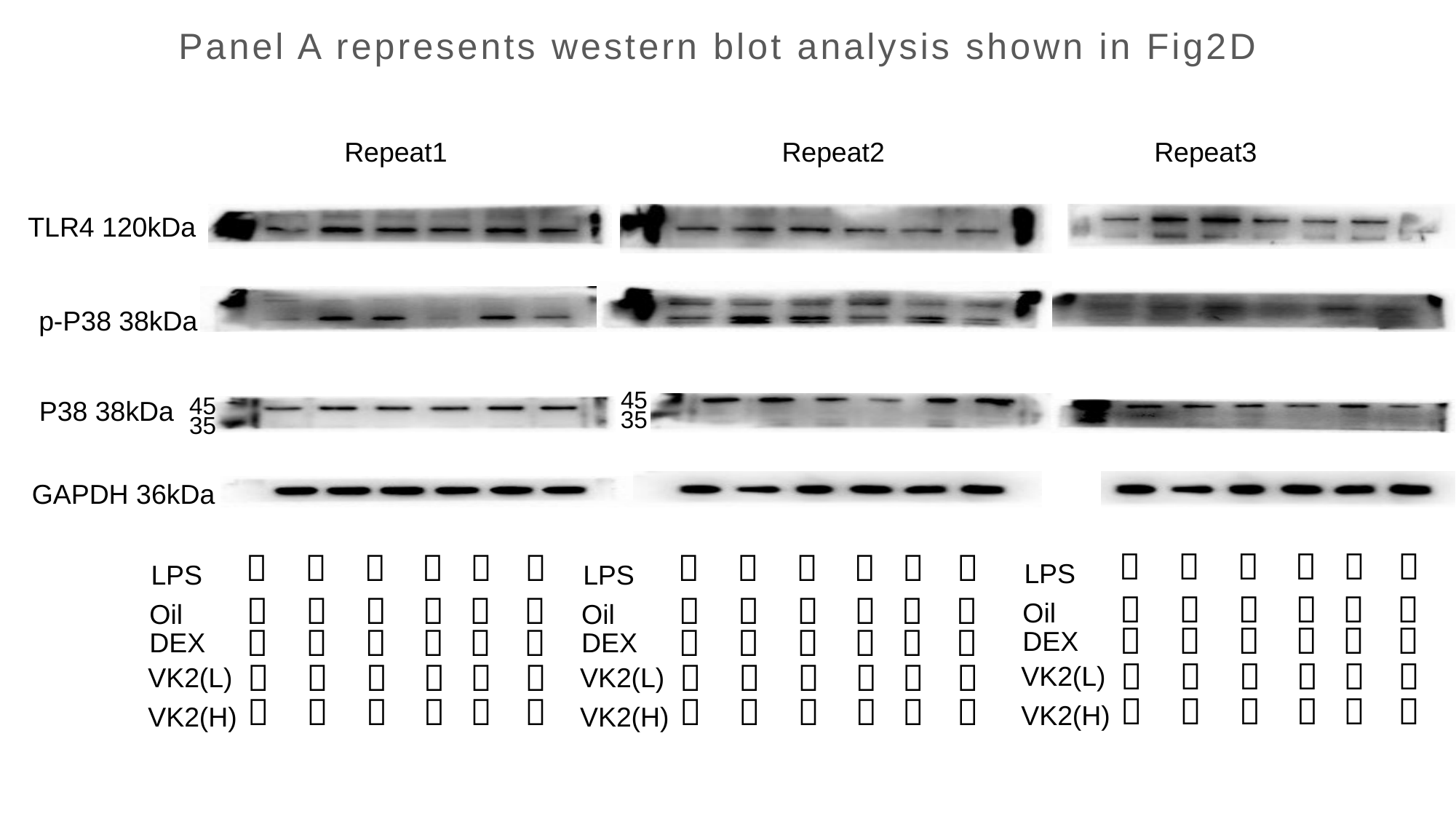

Panel A represents western blot analysis shown in Fig2D
Repeat1
Repeat2
Repeat3
TLR4 120kDa
p-P38 38kDa
45
45
P38 38kDa
35
35
GAPDH 36kDa
－
＋
＋
＋
＋
＋
－
＋
＋
＋
＋
＋
－
＋
＋
＋
＋
＋
LPS
LPS
LPS
－
－
＋
－
－
－
－
－
＋
－
－
－
－
－
＋
－
－
－
Oil
Oil
Oil
－
－
－
＋
－
－
－
－
－
＋
－
－
－
－
－
＋
－
－
DEX
DEX
DEX
－
－
－
－
＋
－
－
－
－
－
＋
－
－
－
－
－
＋
－
VK2(L)
VK2(L)
VK2(L)
－
－
－
－
－
＋
－
－
－
－
－
＋
－
－
－
－
－
＋
VK2(H)
VK2(H)
VK2(H)

## Slide 2
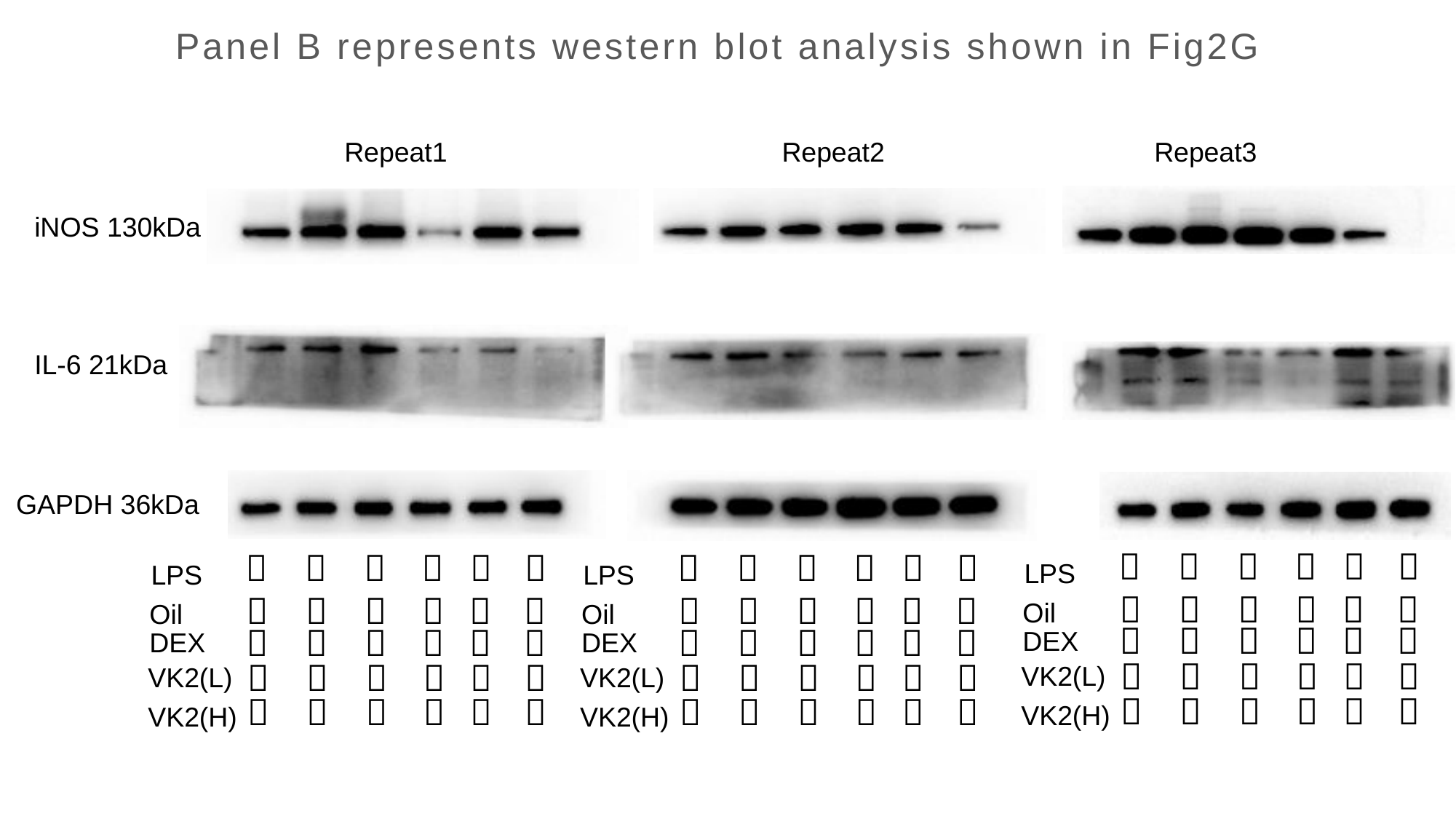

Panel B represents western blot analysis shown in Fig2G
Repeat1
Repeat2
Repeat3
iNOS 130kDa
IL-6 21kDa
GAPDH 36kDa
－
＋
＋
＋
＋
＋
－
＋
＋
＋
＋
＋
－
＋
＋
＋
＋
＋
LPS
LPS
LPS
－
－
＋
－
－
－
－
－
＋
－
－
－
－
－
＋
－
－
－
Oil
Oil
Oil
－
－
－
＋
－
－
－
－
－
＋
－
－
－
－
－
＋
－
－
DEX
DEX
DEX
－
－
－
－
＋
－
－
－
－
－
＋
－
－
－
－
－
＋
－
VK2(L)
VK2(L)
VK2(L)
－
－
－
－
－
＋
－
－
－
－
－
＋
－
－
－
－
－
＋
VK2(H)
VK2(H)
VK2(H)

## Slide 3
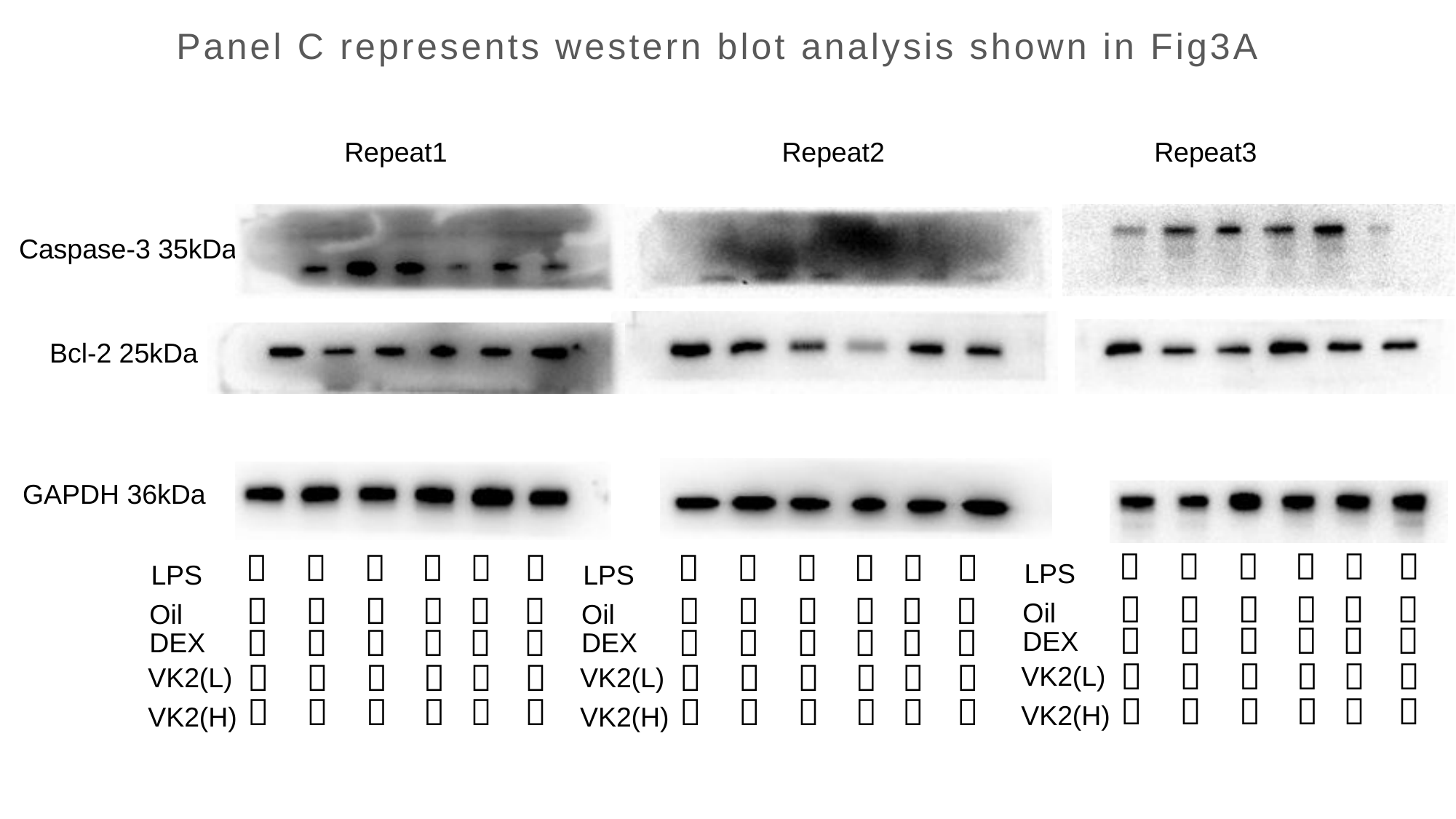

Panel C represents western blot analysis shown in Fig3A
Repeat1
Repeat2
Repeat3
Caspase-3 35kDa
Bcl-2 25kDa
GAPDH 36kDa
－
＋
＋
＋
＋
＋
－
＋
＋
＋
＋
＋
－
＋
＋
＋
＋
＋
LPS
LPS
LPS
－
－
＋
－
－
－
－
－
＋
－
－
－
－
－
＋
－
－
－
Oil
Oil
Oil
－
－
－
＋
－
－
－
－
－
＋
－
－
－
－
－
＋
－
－
DEX
DEX
DEX
－
－
－
－
＋
－
－
－
－
－
＋
－
－
－
－
－
＋
－
VK2(L)
VK2(L)
VK2(L)
－
－
－
－
－
＋
－
－
－
－
－
＋
－
－
－
－
－
＋
VK2(H)
VK2(H)
VK2(H)

## Slide 4
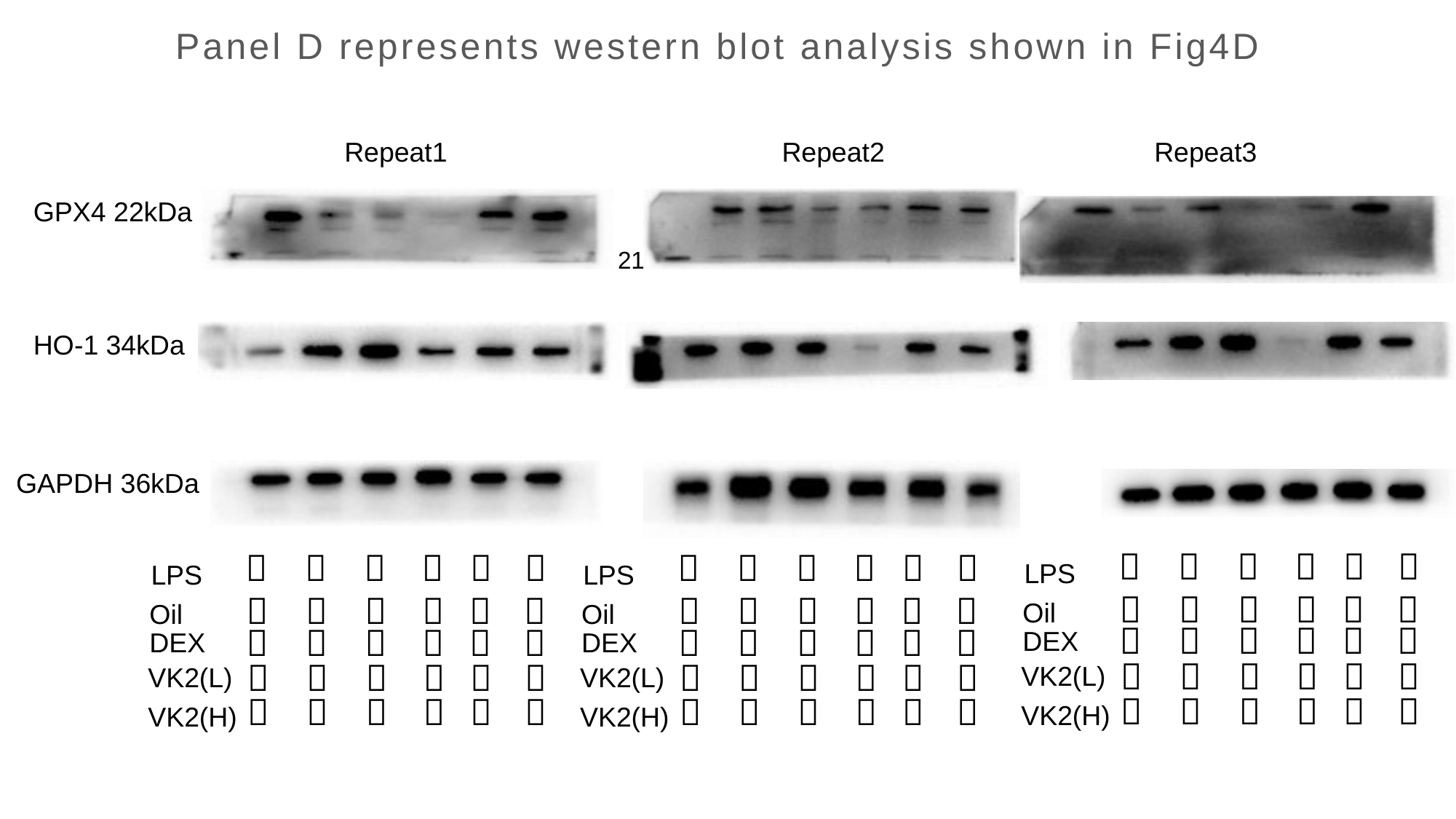

Panel D represents western blot analysis shown in Fig4D
Repeat1
Repeat2
Repeat3
GPX4 22kDa
21
HO-1 34kDa
GAPDH 36kDa
－
＋
＋
＋
＋
＋
－
＋
＋
＋
＋
＋
－
＋
＋
＋
＋
＋
LPS
LPS
LPS
－
－
＋
－
－
－
－
－
＋
－
－
－
－
－
＋
－
－
－
Oil
Oil
Oil
－
－
－
＋
－
－
－
－
－
＋
－
－
－
－
－
＋
－
－
DEX
DEX
DEX
－
－
－
－
＋
－
－
－
－
－
＋
－
－
－
－
－
＋
－
VK2(L)
VK2(L)
VK2(L)
－
－
－
－
－
＋
－
－
－
－
－
＋
－
－
－
－
－
＋
VK2(H)
VK2(H)
VK2(H)
