# Supplementary material for: Vitamin K2 (MK-7) attenuates LPS-induced acute lung injury via inhibiting inflammation, apoptosis, and ferroptosis
Source: PLoS One. 2023 Nov 27;18(11):e0294763. doi: 10.1371/journal.pone.0294763 (PMC10681318; doi:10.1371/journal.pone.0294763)
Supplement: Attachment — Submitted filename: PONE-D-23-23562 Response to Reviews.docx [file pone.0294763.s002.docx]

Point-by-Point Response to the comments raised by Reviewers

**Journal name:** PLOS ONE

**Article ID:** PONE-D-23-23562

**Manuscript Title:** Vitamin K2 (MK-7) attenuates LPS-induced acute lung injury via inhibiting inflammation, apoptosis, and ferroptosis

**Authors:** Yulian Wang, Weidong Yang, Lulu Liu, Lihong Liu, Jiepeng Chen, Lili Duan, Yuyuan Li, Shuzhuang Li

Dear Editor:

First, I would like to express my deep gratitude to you and to the reviewers for the constructive comments and suggestions. We have completely revised the manuscript and addressed all the concerns raised by the reviewers.

A Point-by-Point Response to the editor’s and reviewers’ comments is given in the following pages. We hope that the revised manuscript can be accepted by PLOS ONE.

Sincerely

Yuyuan Li, Ph.D.

E-mail: [liyuyuan831221@163.com](mailto:liyuyuan831221@163.com)

Advanced Institute for Medical Sciences, Dalian Medical University

Tel.: +86 411 8611 8983. **Response to editor:**

Q1: Please ensure that your manuscript meets PLOS ONE's style requirements, including those for file naming. The PLOS ONE style templates can be found at

https://journals.plos.org/plosone/s/file?id=wjVg/PLOSOne_formatting_sample_main_body.pdf and

https://journals.plos.org/plosone/s/file?id=ba62/PLOSOne_formatting_sample_title_authors_affiliations.pdf

**Response:** The authors appreciate this comment very much. Following this suggestion, we **have modulated** our manuscript to meet PLOS ONE’s style requiremnts.

Q2: We noticed you have some minor occurrence of overlapping text with the following previous publication(s), which needs to be addressed:

- https://www.frontiersin.org/articles/10.3389/fphar.2021.790072/full

- https://www.mdpi.com/2072-6643/13/12/4441/htm

- <https://pubmed.ncbi.nlm.nih.gov/36002086/>

In your revision ensure you cite all your sources (including your own works), and quote or rephrase any duplicated text outside the methods section. Further consideration is dependent on these concerns being addressed.

**Response:** The authors agree to the opinion from the reviewer completely. Following this suggestion, we **have reedited** the sections of Introduction and Discussions in our revised manuscript (**please see Pages 5 and 15, marked in red**).

Q3: PLOS ONE now requires that authors provide the original uncropped and unadjusted images underlying all blot or gel results reported in a submission’s figures or Supporting Information files. This policy and the journal’s other requirements for blot/gel reporting and figure preparation are described in detail at https://journals.plos.org/plosone/s/figures#loc-blot-and-gel-reporting-requirements and https://journals.plos.org/plosone/s/figures#loc-preparing-figures-from-image-files. When you submit your revised manuscript, please ensure that your figures adhere fully to these guidelines and provide the original underlying images for all blot or gel data reported in your submission. See the following link for instructions on providing the original image data: https://journals.plos.org/plosone/s/figures#loc-original-images-for-blots-and-gels.

In your cover letter, please note whether your blot/gel image data are in Supporting Information or posted at a public data repository, provide the repository URL if relevant, and provide specific details as to which raw blot/gel images, if any, are not available. Email us at plosone@plos.org if you have any questions.

**Response:** The authors appreciate this comment very much. The original western blot images were shown as below and in the supplementary materials. Due to the limited amount of the protein samples and to avoid the effects of the stripping procedures, we did have cut the blots, however, the molecular weights for the proteins are all as accurate as we expected.

Q4: Please include your full ethics statement in the ‘Methods’ section of your manuscript file. In your statement, please include the full name of the IRB or ethics committee who approved or waived your study, as well as whether or not you obtained informed written or verbal consent. If consent was waived for your study, please include this information in your statement as well.

**Response:** The authors appreciate this comment very much. The experiment was conducted with the approval of the Dalian Medical University Animal Care and Use Committee (AEE20051). We **have uploaded** the **Ethical Approval** as a supplementary material.

**Response to Reviewer 1:**

Q1: As we all know, the COVID-19 pandemic is a research hotspot. Because of this, many researchers are trying to grasp this hotspot. But a lot of the research has nothing to do with COVID-19. The current study can only be said to focus on the relationship between Vitamin K2 and LPS-induced acute lung injury, and the relationship with COVID-19 is minimal. However, the authors introduce too much COVID-19 in the Abstract and Introduction. The authors must be aware of this and make the necessary corrections. There is no need for researchers to rub hot spots.

Lipopolysaccharide (LPS) can indeed cause acute lung injury (ALI) or acute respiratory distress syndrome (ARDS). The symptoms of ALI induced by LPS and those of a new coronavirus infection may share some similarities, but there are important distinctions between the two conditions.

**Response:** The authors agree to the opinion from the reviewer completely. Following this suggestion, we **have removed** some contents about COVID-19 in the Abstract and Introduction, such as “and a common global cause of death due to COVID-19 in recent years”, “The Coronavirus Disease 2019 (COVID-19) pandemic caused by the severe acute respiratory syndrome type 2 Coronavirus (SARS-CoV-2) has plunged the world into an unprecedented health, social, and economic crisis [1].”, “are committed steps and lead to high morbidity and mortality during the development of COVID-19 infection”, and “In the LPS-induced ALI model, mice showed similar pathological damage to COVID-19 [4].” (**please see the revised manuscript**).

Q2: Lines 76 and 77: This seemingly straightforward sentence is an essential basis for your research, but unfortunately, this reference cannot support this argument. Please carefully check this citation.

**Response**: The authors appreciate this comment very much. Because this sentence is about COVID-19, so we **have deleted** it (**please see Page 5 in the revised manuscript**).

Q3: Line 131: How did the authors choose this exposure dose? Did the mice die at this dose? What is the mortality rate?

**Response**: The authors acknowledge the reviewer’s comment and suggestion very much. Some studies found the intervention dose of VK2 for disease was between 15 to 60mg/kg [1-3]. However, there is no unified standard for the recommended intake of VK2 in various countries. The body surface area method was used for conversion, corresponding to 7.2-15.6 ug/kg for mice based on the recommended range is 55-120 μg/d [4,5]. Therefore, 0.2 and 15 mg/kg doses of VK2 were used in formal experiments, in order to further investigate the optimal dose of VK2 for mice with ALI. The part was added into the Discussion section: “We determined the VK2 intervention dose for this experiment based on previous studies [18,19,25] and on the range of recommended VK2 intakes in each country [26,27].” (**please see Page 15, marked in red**)

References:

[1] Hu S, Ma Y, Xiong K, Wang Y, Liu Y, Sun Y, Yang Y, Ma A. Ameliorating Effects of Vitamin K2 on Dextran Sulfate Sodium-Induced Ulcerative Colitis in Mice. Int J Mol Sci. 2023 Feb 3;24(3):2986. doi: 10.3390/ijms24032986.

[2] Jin C, Tan K, Yao Z, Lin BH, Zhang DP, Chen WK, Mao SM, Zhang W, Chen L, Lin Z, Weng SJ, Bai BL, Zheng WH, Zheng G, Wu ZY, Yang L. A Novel Anti-Osteoporosis Mechanism of VK2: Interfering with Ferroptosis via AMPK/SIRT1 Pathway in Type 2 Diabetic Osteoporosis. J Agric Food Chem. 2023 Feb 15;71(6):2745-2761. doi: 10.1021/acs.jafc.2c05632.

[3] El-Sherbiny M, Atef H, Helal GM, Al-Serwi RH, Elkattawy HA, Shaker GA, Said E, Abulfaraj M, Albalawi MA, Elsherbiny NM. Vitamin K2 (MK-7) Intercepts Keap-1/Nrf-2/HO-1 Pathway and Hinders Inflammatory/Apoptotic Signaling and Liver Aging in Naturally Aging Rat. Antioxidants (Basel). 2022 Oct 30;11(11):2150. doi: 10.3390/antiox11112150.

[4] Simes DC, Viegas CSB, Araújo N, Marreiros C. Vitamin K as a Diet Supplement with Impact in Human Health: Current Evidence in Age-Related Diseases. Nutrients. 2020 Jan 3;12(1):138. doi: 10.3390/nu12010138.

[5] Marles RJ, Roe AL, Oketch-Rabah HA. US Pharmacopeial Convention safety evaluation of menaquinone-7, a form of vitamin K. Nutr Rev. 2017 Jul 1;75(7):553-578. doi: 10.1093/nutrit/nux022.

Q4: Lines 269 to 279: Please see my above comments.

**Response:** The authors appreciate the reviewer’s helpful comments. Following this suggestion, we **have removed** some contents about COVID-19 in the Discussion, such as “Novel coronavirus ALI (COVID-19), caused by SARS-CoV-2, has significant impact on global health and socio-economic development [26], while the current treatments are not effective in treating ALI.” and “And it is also a major factor triggering the further development of COVID-19, which is highly consistent with ALI [29].” (**please see Page 15 in the revised manuscript**).

Q5: Lines 351 to 353: This reviewer does not think this sentence could be listed in the Conclusion Section. The authors should never forget that you only injected LPS intraperitoneally into mice and did not artificially infect experimental animals with the new coronavirus.

**Response:** The authors appreciate the reviewer’s helpful comments. Following this suggestion, we **have replaced** “COVID-19” with “ALI” (**please see Page 18, marked in red**).

**Response to Reviewer 2:**

Q1: Due to its multiple pharmacological properties, VK2 has become a research hotspot for scientists in recent years, including anti-inflammatory, anti-oxidant, anti-apoptotic, and anti-ferroptosis properties. According to this study, VK2 protects against ALI caused by LPS by suppressing the P38 MAPK signaling pathway and ferroptosis. Overall, the paper is well-written, but a few minor revisions are necessary.

**Response:** Thank you for the comment.

Q2: Some abbreviations that appeared in the abstract were not explained, such as "MDA" and "GSH".

**Response:** The authors acknowledge the reviewer’s comment and suggestion very much, which is valuable in improving the quality of our manuscript. Following this suggestion, we **have added** the full name of “MDA” and “GSH” (**Please see Page 2, marked in red**).

Q3: In animal experiments, why is it that the positive control group receives the LPS injection first, while the other groups receive the injection afterward? It is necessary to provide a reasonable explanation for this. Would it be better to replace a positive control group?

**Response**: The authors acknowledge the reviewer’s comment and suggestion very much. Dexamethasone (DEX) is a glucocorticoid widely used for the treatment of ALI. In order to simulate the real situation of clinical medication, mice were administrated DEX after LPS injection. In this study, we evaluated the effect of VK2 pretreatment on ALI. The positive control drug is included in the experiments to allow comparisons between the test sample and well-known drugs. Our results show that the effect achieved by VK2 pretreatment is comparable to that of positive drug treatment.

Q4: Is it necessary to replace "treatment" with "pretreatment" in line 239, line 246 and line 345?

**Response:** The authors appreciate the reviewer’s helpful comments. Following this suggestion, we **have replaced** “treatment” with “pretreatment” in the revised manuscript (**Please see Pages 7, 8, 13, 14, and 18, marked in red**).

Q5: As shown in Figures 4 A, B, and C, the results are not significant and require further investigation.

**Response**: This is a very constructive suggestion, which is valuable in improving the quality of our manuscript. Following this suggestion, we **have readjusted** the statistical analysis in the revised manuscript. After comparing multiple sets of data with significant differences, we used T-test for pairwise comparison. We **have not only modified** Figures 4A,B, and C, but also Figure 2I and Figure 4F. The revised figures are as follows:

Figure 2

Figure 4

Q6: The association between P38 MAPK pathway and Bcl-2 and Caspase-3 needs to be explained in lines 308-310.

**Response**: The authors appreciate this comment very much. Following this suggestion, we **have added** some discussions about the association between P38 MAPK pathway and Bcl-2 and Caspase-3: “Studies have shown that phosphorylated P38 MAPK can activate caspase-3 and promote apoptosis by downregulating Bcl-2 [44-46]” (**Please see Page 16, marked in red**).

Reference:

[44] De Chiara G, Marcocci ME, Torcia M, Lucibello M, Rosini P, Bonini P, Higashimoto Y, Damonte G, Armirotti A, Amodei S, Palamara AT, Russo T, Garaci E, Cozzolino F. Bcl-2 Phosphorylation by p38 MAPK: identification of target sites and biologic consequences. J Biol Chem. 2006 Jul 28;281(30):21353-21361. doi: 10.1074/jbc.M511052200.
[45] Zanotto-Filho A, Braganhol E, Battastini AM, Moreira JC. Proteasome inhibitor MG132 induces selective apoptosis in glioblastoma cells through inhibition of PI3K/Akt and NFkappaB pathways, mitochondrial dysfunction, and activation of p38-JNK1/2 signaling. Invest New Drugs. 2012 Dec;30(6):2252-62. doi: 10.1007/s10637-012-9804-z.
[46] Huang YK, Chang KC, Li CY, Lieu AS, Lin CL. AKR1B1 Represses Glioma Cell Proliferation through p38 MAPK-Mediated Bcl-2/BAX/Caspase-3 Apoptotic Signaling Pathways. Curr Issues Mol Biol. 2023 Apr 13;45(4):3391-3405. doi: 10.3390/cimb45040222.

Q7: The format of the article is not standardized, such as ' Caspase-3 ' in several places without capitalization. It is strongly recommended that you double-check.

**Response**: The authors acknowledge the reviewer’s comment and suggestion very much, which is valuable in improving the quality of our manuscript. We have changed “caspase-3” into “Caspase-3” in the entire manuscript (**Please see Pages 12 and 16, marked in red**).

Q8: The limitations of this experiment were not discussed in the discussion.

**Response:** The authors appreciate the reviewer’s helpful comments. Following this suggestion, we **have added** some discussions about the limitations of this study “Nevertheless, despite widespread adoption, the LPS-induced ALI model may not entirely encompass the complexity and heterogeneity of human ALI. It is essential to bolster animal studies with clinical research and validation. In the future, we will carry out relevant in-depth work to solve this problem.” (**Please see Page 18, marked in red**).
